# High-Efficiency Parallel Cryptographic Accelerator for Real-Time Guaranteeing Dynamic Data Security in Embedded Systems

**DOI:** 10.3390/mi12050560

**Published:** 2021-05-15

**Authors:** Zhun Zhang, Xiang Wang, Qiang Hao, Dongdong Xu, Jinlei Zhang, Jiakang Liu, Jinhui Ma

**Affiliations:** School of Electronic and Information Engineering, Beihang University, Beijing 100191, China; microzhun@buaa.edu.cn (Z.Z.); haoqiang1994@buaa.edu.cn (Q.H.); xudongdong1994@buaa.edu.cn (D.X.); zhangjinlei@buaa.edu.cn (J.Z.); ljiakang@buaa.edu.cn (J.L.); sy2002514@buaa.edu.cn (J.M.)

**Keywords:** cryptographic accelerator, dynamic data security, AES-GCM, hardware security, SoC

## Abstract

Dynamic data security in embedded systems is raising more and more concerns in numerous safety-critical applications. In particular, the data exchanges in embedded Systems-on-Chip (SoCs) using main memory are exposing many security vulnerabilities to external attacks, which will cause confidential information leakages and program execution failures for SoCs at key points. Therefore, this paper presents a security SoC architecture with integrating a four-parallel Advanced Encryption Standard-Galois/Counter Mode (AES-GCM) cryptographic accelerator for achieving high-efficiency data processing to guarantee data exchange security between the SoC and main memory against bus monitoring, off-line analysis, and data tampering attacks. The architecture design has been implemented and verified on a Xilinx Virtex-5 Field Programmable Gate Array (FPGA) platform. Based on evaluation of the cryptographic accelerator in terms of performance overhead, security capability, processing efficiency, and resource consumption, experimental results show that the parallel cryptographic accelerator does not incur significant performance overhead on providing confidentiality and integrity protections for exchanged data; its average performance overhead reduces to as low as 2.65% on typical 8-KB I/D-Caches, and its data processing efficiency is around 3 times that of the pipelined AES-GCM construction. The reinforced SoC under the data tampering attacks and benchmark tests confirms the effectiveness against external physical attacks and satisfies a good trade-off between high-efficiency and hardware overhead.

## 1. Introduction

Modern embedded systems are gaining popularity in numerous security-sensitive sectors with great intrinsic reliability, high performance, and good functional adaptation, which are ideal control platforms for executing intensive real-time data processing tasks, such as in automotive, aerospace, avionic, and railway systems. The widespread usages of embedded systems in safety-critical fields have made hardware system security as a prominent issue. Furthermore, the growing demands of embedded systems are pushing System-on-Chip (SoC) towards the dramatic improvements in performance and multiple function; these welcome upswings are inevitably accompanied by the various forms of emerging security threats [1]. Generally, the diversiform threats mainly arise from the untrusted IPs [2], vulnerable firmware and software [3], and even insecure communications with the other devices [4], which are the critical factors on damaging the security of embedded systems in safety-critical applications. During operation, processors are at the heart of embedded systems: attackers can exploit hostile data to trick the internal interpreter into executing unintended commands or accessing unauthorized data, and then they can get the confidential information of system and control the behavior of programs to perform malicious actions. Therefore, the security assurance of the modern embedded system involves a number of challenges; meanwhile, it is worth receiving greater attention among hardware architecture designers.

Information leakages and function failures have been emerging as primary manifestations of being attacked in process-intensive platforms, where the confidentiality and dependability of embedded systems must be ensured. In addition, it also must be admitted that the hardware system protections are not particularly comprehensive due to malicious attacks that may arise from different-level sources, mainly including hardware-level attack and system-level attack. As the hardware-level attack, a malicious logic (well-known as hardware Trojan) hidden in internal logic might be premeditatedly designed to cause the program execution failures at key points or could create the backdoors for both confidential information leakages and subsequent system hijacking to attackers [5]. On the other hand, the external physical attacks can exploit the three invasive methods of bus monitoring, offline analysis, and data tampering to steal the sensitive information and/or activate the built-in hardware Trojan to disorganize the program execution through external access interfaces [6,7]. System-level attacks mainly exploit the security vulnerabilities or bugs in software applications to disturb the instruction executions or cause buffer overflows by injecting malicious codes for subverting the system trustworthiness and obtaining the unauthorized control of system. Taking the stack-smashing attack as an example, we can see that this system-level attack caused by the untrusted program exploits buffer overflow vulnerabilities to take control over attacked operating systems and hardware platforms. Fortunately, various techniques have been developed to defend against stack smashing attacks, such as Write XOR eXecution (W⊕X) [8], Data Execution Prevention (DEP) [9], Address Space Layout Randomization (ASLR) [10], in-stack canaries [11], and some software code integrity checkers [12,13].

In response to both hardware-level and system-level attacks, several security mechanisms have been proposed to protect program executions and control flow behaviors of embedded system, in which, a great majority of the protection techniques try to modify the instruction set architectures and compilers, which make them very hard to be transplanted into diverse embedded system platforms. Furthermore, the widely used system security solutions are based on anti-virus software, which makes it difficult to prevent the anti-virus software’s own vulnerabilities. Previous reports about the integrity protections of program code and control flow according to the program segments, such as transfer functions [14] and basic blocks [15], are considered to be the key marker of the system safety status. However, the run-time data in the main memory of the embedded system is another important factor for secure program executions. Attackers can exploit the data in the stack and heap segment to change the program behavior of the original intention, so that the confidentiality of system dynamic data is critically important. In addition, a few schemes are presented to protect the security of data processing in embedded systems since it is harder to monitor the dynamic data status during program execution without causing significant speed degradation. Moreover, the rapid growth of detection and analysis technologies make it easier for a sophisticated attacker to obtain physical access and launch physical attacks on the insecure off-chip main memory. Attackers can monitor the address/data bus and off-line analysis, and then tamper and inject or replay memory blocks while the program is loading the data to processor; these may result in confidential information leakages, changes of program control flow, and destruction of data block.

In this paper, we proposed a hardware-assisted four-parallel AES-GCM cryptographic accelerator to provide real-time security monitoring and authenticated encryption. Considering the possible confidential data leakages and physical tampering attacks aiming at the main memory, the cryptographic accelerator can protect the confidentiality and integrity of data at run-time and monitor the malicious data tampering injections. During program execution, the D-Cache line data blocks that need to be stored in memory are encrypted and tagged through AES-GCM accelerator and verify integrity Tag to prevent the system data blocks being corrupted or tampered from external memory. An optimal four-parallel AES-GCM hardware architecture achieves the high-efficiency data block encryption while D-Cache keeps consistent with the main memory, which will not cause processor significant speed degradation. The evaluation experiments of running ten different benchmarks have verified the system low performance overhead. The fault injection attacks have confirmed the monitoring effectiveness of cryptographic accelerator. Finally, the resource consumption of SoC is presented and is reasonable in real applications. The specific contributions of this article are as follows:

- An optimal four-parallel AES-GCM hardware architecture is constructed to provide confidentiality and integrity protections for the data at run-time, any unauthorized changes of ciphertext and signature in memory will be detected by authenticating the integrity Tag.
- The dynamic encryption mechanism is achieved according to the D-Cache hit status for the purpose of significantly reducing the system performance overhead induced by cryptographic accelerator.
- Evaluation experiments of four-parallel cryptographic accelerator in terms of performance overhead, security capability, processing efficiency, and resource consumption confirm the SoC effectiveness against external physical attacks and reaches a good trade-off between security capability and hardware overhead.
- The four-parallel hardware accelerator is around 3 times as the pipelined AES-GCM contruction in data encryption efficiency, which will further help reducing performance overhead in encryption and integrity authentication.

The remainder of this paper is organized as follows. Section 2 is dedicated to introducing the SoC assumptions and threat model considered in this work. Section 3 presents the related works concerning design strategies against information leakages and tampering attacks. Section 4 describes the proposed parallel hardware of AES-GCM algorithm in detail. Our proposal SoC hardware architecture is presented in Section 5. Section 6 presents experiments and results of performance overhead, security capability, processing efficiency, and resource consumption. The paper is concluded in Section 7.

## 2. Assumptions and Threat Model

**Figure 1:**
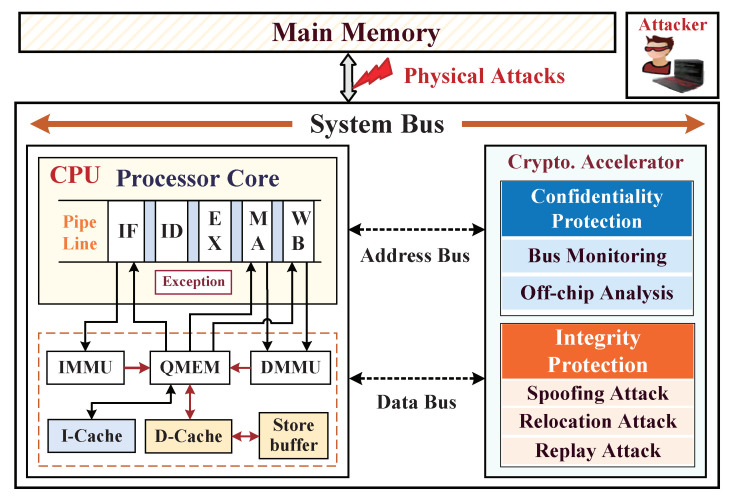
The overall System-on-Chip (SoC) framework with integrating a cryptographic accelerator for dynamic data monitoring against external physical attacks.

Before developing a hardware-assisted cryptographic accelerator for target embedded system platform, its specific trustworthy assumption and target threat model should first be determined, and the associate assumptions of design components (including IP entities) should be classified as trustworthy and untrustworthy. Our SoC framework architecture with integration of a cryptographic accelerator for dynamic data monitoring is shown in Figure 1. We assume that a trustworthy system is a black-box integration of processor core, instruction cache (I-Cache), data cache (D-Cache), system bus, cryptographic accelerator, and common I/O interfaces. The components have been tested earlier, and it has been validated that wherever there are no potential hardware Trojans inserted within internal control logics, and all the adversaries, one cannot tamper the pipeline, registers, I/D-Caches, and control signals inside the embedded processor due to the lack of access channel. We make the boundary assumption regarding the SoC hardware architecture that apart from the on-chip trusted domain, the whole off-chip domain is the untrustworthy region in which peripheral devices may include the main memory, the direct memory access controller (DMAC), the external master, and common hardware blocks. In particular, the off-chip main memory is a weak part, and as it is untrusted, the adversaries can exploit the external data bus to probe confidential information and then access memory to launch physical tampering attacks with malicious data injections.

Our architecture-level solution integrates a hardware-assisted cryptographic accelerator to provide real-time dynamic data protections on defending both confidential information leakages and data tampering attacks. The hardware accelerator integrates a standardized symmetric encryption engine and a hash algorithm engine to ensure the confidentiality and integrity of data exchange and storage at run time. In practice, we make an assumption that the attackers mainly exploit three invasive methods of bus monitoring, offline analysis, and data tampering to steal sensitive data and disorganize the program execution by the external abnormal access. We aim to address the above-mentioned three types of external physical attacks to achieve confidentiality and integrity guarantees of dynamic data provided by the high-efficiency cryptographic accelerator, without it incurring significant performance overhead.

## 3. Preliminaries

Our studies focus on obtaining a prompt identification of the abnormal behaviors through the cryptographic accelerator for avoiding control system malfunction caused by physical attacks. This section introduces the basics of security strategies against confidential information leakages and data tampering measures and then presents the cryptographic algorithm model and activated mechanism to establish the system architecture.

### 3.1. Security Policies against Sensitive Information Leakages

The sensitive information leakages from embedded architecture we considered mainly originate from the external physical attacks aiming at the interface of main memory. In response to external physical threats, the cryptographic algorithm requires a high-speed hardware to complete data encryption and decryption operations that does not incur significant performance overhead. The state-of-the-art schemes of data security protections are mainly based on the three cryptographic methods: confidentiality scheme, integrity scheme, and authentication of signature.

#### 3.1.1. Confidentiality Protection Scheme

Several symmetric-key and asymmetric-key cryptographic algorithms have been proposed to provide the confidentiality, integrity, and authentication (CIA) protections against information leakages. The advanced encryption standard (AES) is a representation of symmetric-key algorithm, which is the block cipher cryptosystem, and its round function encryption consists of SubBytes, ShiftRows, MixColumns, and AddRoundKey; supports the input and output data blocks at the same length of 128 bits with the self-determined key lengths of 128 bits (with 10 rounds), 192 bits (with 12 rounds), and 256 bits (with 14 rounds); and requires different lengths to be designed for hardware implementations according to the required security strength. The AES block-cipher method has a great security capability in confidentiality protection, while the reported stream cipher also can provide very good security features on the data encryption [16]. Moreover, the asymmetric-key Rivest-Shamir-Adleman (RSA) is a high-quality public key cryptographic algorithm, which is an ideal selection for digital signature, key exchange, etc., in large set of security protocols for data exchange [17], but it is expensive compared to symmetric cryptography on computationally intensive tasks, such as applied in the data block encryption.

#### 3.1.2. Integrity Protection Scheme

Although several cryptographic algorithms enhancing data confidentiality have been implemented for the embedded systems, it is still possible for adversaries to crack the AES encrypted information and recover the 16-byte secret key by using the side-channel attacks and then injects tampered data. To further improve the security of encrypted information, the cryptographic hash function is applied as an integrity-protection algorithm that can quickly transform a given amount of information into a fixed number of digital signatures, and then the receiver can authenticate the digital signature to guarantee that the information has not been modified. For instance, a reported study [18] proposed a lightweight hash function named the LHash algorithm to perform the integrity verification of sensitive information at different lengths. The LHash algorithm employs the kind of Feistel-PG structure in the internal permutation for utilizing the permutation layers on nibbles to improve diffusion speed. Another sophisticated hash algorithm is called the GHash function [19]; by employing authenticated encryption mode of Galois/Counter Mode (GCM), it has a great advantage in the high-speed parallel computing to provide data flow integrity authentication with minimum latency and performance overhead, while its implementation is accompanied by a higher hardware complexity.

#### 3.1.3. Authentication of Data Signature

Advancements in security have been achieved by incorporating the cryptographic algorithm and hash function; the authentication of data signature provides an evidence that the confidential information has not been modified during transmission, where the digital signature generated from the synthesis of ciphertext, secret key, and timestamp offer a high confidence in authenticity.

### 3.2. Data Tampering Attacks on External Main Memory

Aiming at embedded processor platforms, a lot of attention has been attracted to the analysis of physical attack methods since they will directly determine the corresponding protection techniques. The well-known side-channel attacks are utilized to obtain cipher keys by observing leakages of power consumption, electromagnetic emissions, or noise [20]. Simple power analysis (SPA) and differential power analysis (DPA) are the frequently-used methods to obtain the cryptographic algorithm power signatures, then lead to the cipher key extractions [21]. A similar solution also works with electromagnetic emissions [22] instead of analyzing the power signature; the chip electromagnetic signature is studied like DPA, and the ciphering key is extracted. Indeed, it is not possible to address all the embedded system security issues in one proposition. In particular, the main memory in embedded system architecture is a weak part: the attacker can easily probe the bus interface between the processor and main memory to obtain confidential data while launching physical tampering attacks on the data bus of the main memory with tampered data injections. Therefore, the predictive analysis of data tampering attacks is essential for designer to guarantee data processing security in embedded systems.

**Figure 2:**
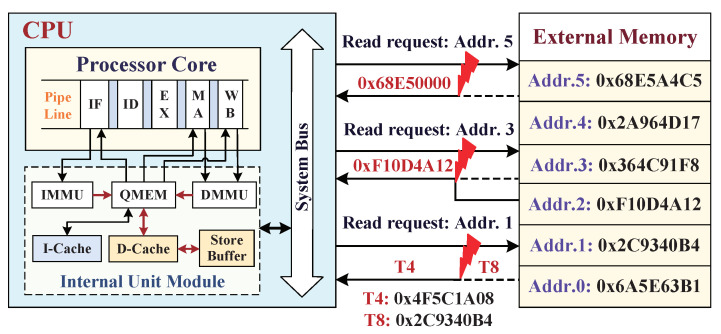
Implemented methods of three types of tampering attacks from main memory: spoofing attack, relocation attack, and replay attack.

In order to better understand the potential data tampering threats, the hardware architecture suffering the tampering attacks from external memory is shown in Figure 2, the framework of which is a brief collection of three types of attacks: the spoofing attack, which utilizes a partially changed data value to impersonate the legitimate data by replacing the valid data block corresponding to the read-back request Address 5, causing the embedded system into malfunction; the relocation attack, which occurs as read-back request Address 3, when one should returned data in Address 3 that are swapped artificially by the data of memory another piece location, such as Address 2; the replay attack, which exploits one registered memory datum targeted at a time *T4* to replay the required data at read-request time *T8*. Compared with the frontal spoofing attack, the relocation attack has an advantage of tricking the processor into accessing unauthorized data, because external memory is encrypted with same scheme, and the attacker will be able to modify and control the processor execution by swapping some encrypted values. In addition, the replay attack at different time can easily overcome the protection against the relocation attacks to modify the processor behavior. Considering the limitations of existing approaches in preventing multiple types of data tampering attacks, the digital signature with its associated data are stored synchronously in memory is a sagacious security strategy.

### 3.3. AES-GCM Model and Activated Mechanism

**Figure 3:**
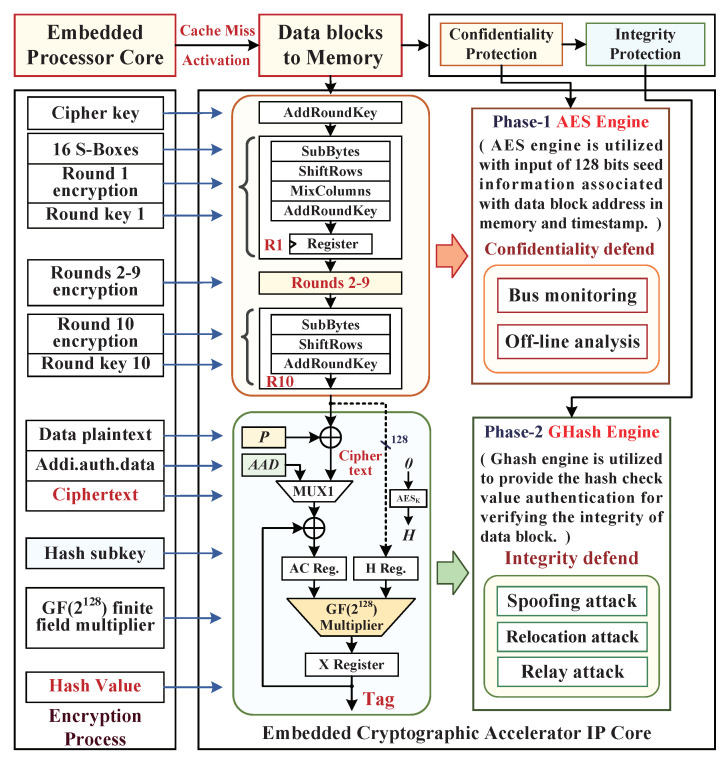
The AES-GCM cryptographic algorithm model for data encryption.

This paper plan adopts AES-GCM algorithm as the embedded cryptographic accelerator IP core and integrates it into the SoC architecture. As shown in Figure 3, we designed the cryptographic accelerator, which consists of two encryption phases of AES engine and Ghash engine. In these, the pipelined AES engine is specified as the confidentiality protection module, which is utilized with an input of 128 bits seed information that is associated with the physical address and timestamp corresponding to the data block; its 1–9 rounds encryption processes are duplicated, and with the round 10 without the *MixColumns* transformation. In addition, the Ghash engine is implemented to provide the hash value authentication for verifying the integrity of the data block, which, through reading the XORed value of data plaintext and AES encrypted physical address (key stream), then encrypts the input plaintext data block and computes an authenticated Tag through the construction of Galois field $GF(2^{128})$ multiplier. This Ghash engine based on block cipher encryption has fixed input parameters, where the hash subkey (*H*) is generated by directly applying the AES engine to encrypt the 128-bit zero block and then as the Ghash engine input subkey *H*.

Hardware-assisted AES-GCM cryptographic accelerator efficiently extends achieving the high-efficiency data processing speed with a reasonable resource overhead. However, performing the encryption and decryption operations on all stored data is unacceptable on processor executing intensive data processing tasks, since excessively encrypted data block protections will cause a large performance overhead to processor program execution. Combined with the superiority of AES engine and Ghash engine on confidentiality and integrity protections, the dynamic data monitoring mechanism is an outstanding technique that works by activating the security IP core only when the D-Cache addressing misses (under the Write-Back mechanism). Making full use of the locality principle of D-Cache on mapping the main memory, we adopted the Write-Back mechanism in memory access, which does not write data blocks to the main memory synchronously when CPU writes to the D-Cache (D-Cache hit); here, the D-Cache values are inconsistent with the data in the main memory, such that external attacks can not cause target SoC information leakages or function failure before the main memory is overwritten. If D-Cache misses, the central processing unit (CPU) will directly access the main memory and write back according to the access address, while the D-Cache will keep consistent with the main memory. In this consistent process, many data blocks from D-Cache will write back to the main memory at one time with AES-GCM hardware accelerator encryption.

## 4. Parallel Hardware Architecture Based on AES-GCM

In this section, we present the details of the parallel hardware architecture implementation based on the AES-GCM algorithm, which contains four main components: the hardware structure of AES encryption, the optimized composite field implementation of S-Boxes, Galois/Counter mode of Galois Hash, and AES-GCM parallel architecture.

### 4.1. Hardware Multiplexing Structure of AES Encryption

**Figure 4:**
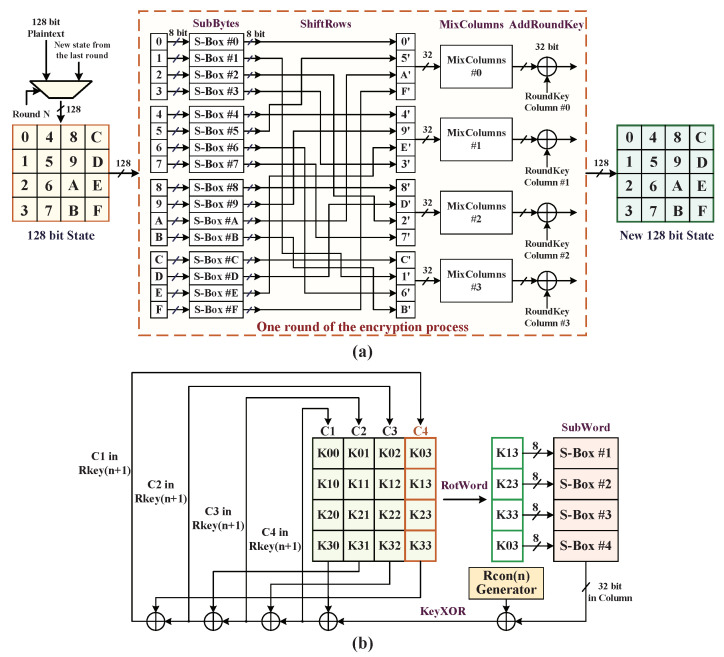
Hardware multiplexing structure of AES encryption algorithm: (**a**) round encryption process; (**b**) key expansion process.

We employed the AES-128 encryption algorithm as the symmetric key block cipher cryptosystem engine. Considering the AES-GCM cryptographic accelerator in terms of speed, area, and power consumption, the rollback multiplexing structure of AES engine is utilized to complete the 10 rounds encryption and the key expansion process, as illustrated in detail in Figure 4. In initial round, the 128 bits state array is generated with the input bitwise XOR values of 128 bits plaintext and cipher key and then begins to execute one round of the encryption process consisting of the four transformations of *SubBytes*, *ShiftRows*, *MixColumns*, and *AddRoundKey*. In this context, *SubBytes* is a non-linear function that substitutes all the bytes of state array from a lookup table of 16 × 16 bytes array, named S-Box; *ShiftRows* is completed by using hardware dislocation wire connections of signals; *MixColumns* is realized through a constant matrix and state matrix multiplicative inversion under the irreducible polynomial of $P\left(x\right)=x^{8}+x^{4}+x^{3}+x+1$ over the finite field $GF(2^{8})$ that each byte of a column in state matrix is mapped into a new value; *AddRoundKey* utilizes a state array in column XORed with the expanded key that results from the key expansion process. Including the first round of encryption, two inputs of the multiplexer module are implemented to perform the 10 rounds encryption in the multiplexing structure, where the *MixColumns* transformation is bypassed in the 10th round.

We designed a key expansion hardware structure to represent the main functionality of the key expansion operations, which consists of four subtransforms: *RotWord* takes one 128 bits cipher key as the initial array, which is similar with *ShiftRows* to rotate the column C4 upward one lattice; *SubWord* is similar to *SubBytes*, substituting the 4 bytes of rotational column from the lookup table of S-Box; *RconGen* uses a constant vector generator to create a round constant hexadecimal bytes column “Rcon(*n*), 0, 0, 0”, where 1≤n≤10; *KeyXOR* executes two XOR operations between the first column C1 of cipher key array, S-Box substituted column, and Rcon(1) constant column, and finally replaces the C1 bytes with the new XORed values. Meanwhile, the columns C2, C3, and C4 are replaced with new C3, C4, and C1, respectively, to constitute a new round(*n* + 1) key array after the same transformation scheme. This key expansion module has overcame a large overhead in terms of power consumption and resource utilization and is suitable for the application in target embedded system.

### 4.2. Optimized Composite Field Implementation of S-Boxes

**Figure 5:**
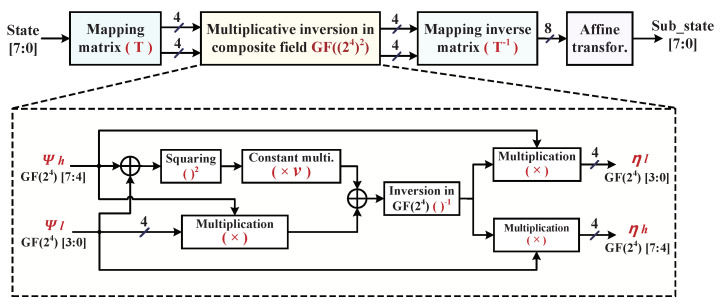
S-Box implementation architecture using normal basis over composite field $GF({\left(2^{4}\right)}^{2})$.

Among the hardware implementation of full AES encryption, the S-Box is known as non-linear transformation in *SubBytes*, which occupies the most hardware resources and consumes around three quarters of its power [23]. Fortunately, the S-box implementation with the composite field arithmetic is an efficient optimization technology to reduce the *SubBytes* hardware realization complexity instead of to look up the table; it is suitable to achieve both low area and low delay in a resource-limited embedded system. For actualizing the S-Box non-linear transformations with input and output bytes in the circuit structure, the irreducible polynomial of $P\left(x\right)=x^{8}+x^{4}+x^{3}+x+1$ is utilized to construct the finite field $GF(2^{8})$. Normally, the S-Box construction can be decomposed into the composite field $GF({\left(2^{4}\right)}^{2})$ that consists of the four stages: isomorphic mapping matrix (***T***), multiplicative inversion, inverse isomorphic mapping matrix ($T^{-1}$), and affine transformation, as shown in Figure 5, in which the isomorphic mapping matrix is utilized to transform the input vector from finite field $GF(2^{8})$ to composite field $GF({\left(2^{4}\right)}^{2})$, the multiplicative inversion calculates the inverse element over $GF(2^{8})$, the inverse isomorphic mapping matrix is used to revert the computing results back to finite field $GF(2^{8})$, and affine transformation outputs the bytes of S-Box. The implementation of AES S-Box based on affine transformation is expressed as the following.

(1) $$S\left(X\right)=AX^{-1}+b=M\times {\left(T\times X\right)}^{-1}+b$$

where ***X*** is an 8-bit input column vector, ***A*** is an 8 × 8 constant binary matrix, $X^{-1}$ is the multiplicative inversion vector over $GF(2^{8})$ corresponding to ***X***, the ***b*** is an artificial 8-bit constant vector, ***M*** is a merged matrix of inverse isomorphic mapping matrix $T^{-1}$ multiply with affine matrix ***A***, as $M=A\times T^{-1}$, and the 8-bit S(***X***) vector is performed after the affine transformation to replay the corresponding elements in S-Box.

Considering the design demands for low-complexity S-Box according to composite field arithmetics, affine transformation can be implemented through XOR operations on data bits, the multiplicative inversion over $GF(2^{8})$ should be decomposed to composite field $GF({\left(2^{4}\right)}^{2})$, and then the multiplicative inversion operations over $GF({\left(2^{4}\right)}^{2})$ are divided into multiplications over $GF(2^{4})$ and multiplicative inversion over $GF(2^{4})$. Figure 5 illustrates the S-Box implementation architecture using normal basis over composite field $GF({\left(2^{4}\right)}^{2})$. To reduce the redundant circuits of S-Box multiplicative inversion realization over the composite field, previous research works have indicated that the common subexpression elimination algorithms are effective to find the best irreducible polynomials and isomorphism mapping [24,25]. We also note that pruned-tree-search strategy is employed to search a family of 432 possible combinations of different isomorphism matrices operations for minimal logic gate consumption and find out the best optimized basis matrix in the reported work [26]. In the best case after the comprehensive comparisons, the following irreducible polynomial over $GF({\left(2^{4}\right)}^{2})$ is selected to obtain the coordinates of multiplicative inversion outputs by using normal basis, as shown the dotted rectangle structure in Figure 5.

(2) $$GF\left({\left(2^{4}\right)}^{2}\right):f\left(u\right)=x^{2}+x+v$$

where the coefficient ***v*** is ${\left(0001\right)}_{2}$. Suppose ψ is an element over finite field $GF(2^{8})$, $\psi_{h},\psi_{l}$∈$GF(2^{4})$; then ψ can be represented by using normal basis as $\psi_{h}$$X^{16}+\psi_{l}$***X***. Therefore, the multiplicative inversion output η equals to $\psi^{-1}$, which can be mapped into composite field $GF({\left(2^{4}\right)}^{2})$ and represented as follows. Its corresponding architecture is illustrated as Figure 5.

(3) $$\eta ={\left(\psi \right)}^{-1}={\left({\left(\psi_{h}+\psi_{l}\right)}^{2}v+\psi_{h}\psi_{l}\right)}^{-1}\times \left(\psi_{h}X^{16}+\psi_{l}X\right)$$

For the optimal resource consideration, we constructed the hardware module of the best case reported in Reference [26] to implement the S-Box. The very compact implementation of S-Box only needs 80 XOR, 34 NAND, and 6 NOR, and it maintains a low critical path delay in target AES engine implementation.

### 4.3. $GF(2^{128})$ Multiplier Implementation of Galois Hash

**Figure 6:**
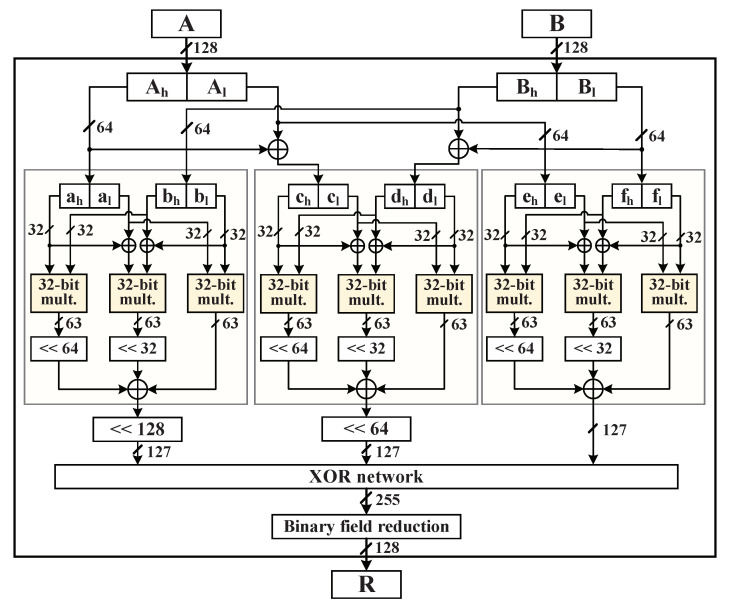
$GF(2^{128})$ multiplier architecture implementation by using the Karatsuba–Ofman multiplier.

After expatiating the 128-bit AES hardware implementation, in the authenticated encryption and decryption procedures combined with Galois/Counter Mode (GCM), the construction of $GF(2^{128})$ multiplier in Ghash engine is the key point in performing the high-speed AES-GCM cryptographic accelerator. We have considered the commonly used $GF(2^{128})$ multiplier algorithms in previous works, such as the Mastrovito multiplier [27] and the Karatsuba-Ofman (KO) multiplier [28]. According to the experiential latency expression $\frac{n}{q}+\log_{2}\left(q\right)$ of implemented architecture, the increasing number of parallel structures (*q*) will result in lower time cycles and a higher throughput during processing the input *n* data blocks. To reduce the hardware complexity of $GF(2^{128})$ multiplier in the Ghash function, we utilized the efficient realization of the KO bit-parallel $GF(2^{128})$ multiplier to keep a lower timing complexity. The $GF(2^{128})$ multiplier architecture implementation by using the KO multiplier is shown in Figure 6.

According to the literature, it is known that the KO algorithm adopts the recursive method to decrease the multiplicative and additive complexities in polynomial multiplication [29]. At the first stage, the *m*-bit inputs *A* and *B* are split evenly into four $\frac{m}{2}$-bit terms of $A_{h}$, $A_{l}$, $B_{h}$, and $B_{l}$ through the KO algorithm, assuming that a(x) and b(x) are two elements in $GF(2^{m})$; both these elements can be represented polynomially as follows.

(4) $$\begin{array}{c} a\left(x\right)=x^{m/2}\left(x^{m/2-1}\cdot a_{m-1}+\cdots +a_{m/2}\right)+\\ \;\;\;\left(x^{m/2-1}\cdot a_{m/2-1}+\cdots +a_{0}\right)=x^{m/2}A_{h}+A_{l}\\ b\left(x\right)=x^{m/2}\left(x^{m/2-1}\cdot b_{m-1}+\cdots +b_{m/2}\right)+\\ \;\;\;\left(x^{m/2-1}\cdot b_{m/2-1}+\cdots +b_{0}\right)=x^{m/2}B_{h}+B_{l} \end{array}$$

Then, the polynomial product R(x)=a(x)·b(x)modg(x) can be represented as follows.

(5) $$\begin{array}{c} R\left(x\right)=x^{m}A_{h}B_{h}+x^{m/2}\left(A_{h}B_{l}+A_{l}B_{h}\right)+A_{l}B_{l}\\ \;\;\;=x^{m}A_{h}B_{h}+x^{m/2}[A_{h}B_{h}+\left(A_{h}+A_{l}\right)\cdot \\ \;\;\;\;\left(B_{h}+B_{l}\right)+A_{l}B_{l}]+A_{l}B_{l} \end{array}$$

Deduced from the Equation (5), the implementation of hardware architecture of $GF(2^{128})$ multiplier is subdivided into nine 32-bit multipliers through two recursions, and every three 32-bit multiplier can perform a 64-bit multiplier module, as depicted in Figure 6. This two-step parallel multiplier of KO algorithm has a good balance between the hardware complexity and computing speed.

### 4.4. AES-GCM Parallel Architecture Mechanism

**Figure 7:**
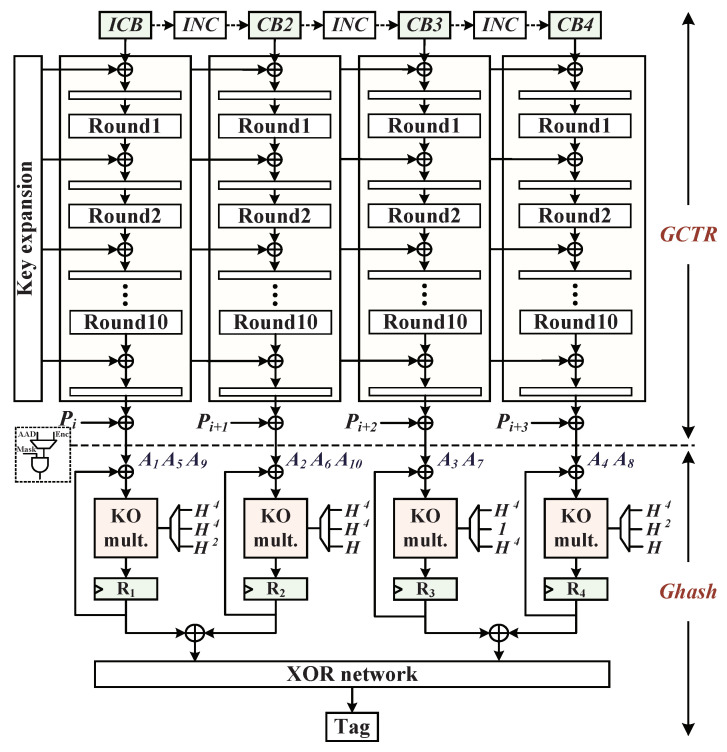
The 4-parallel AES-GCM architecture based on the combination of four AES cores and four KO multipliers.

We have presented the Ghash 128-bit multiplier architecture over the binary Galois field that provides fast hash computation. The proposed AES-GCM hardware architecture is based on the combination of the AES engine and the Ghash engine to support a high rate of data-authenticated encryption, since it can take advantages of the parallel processing technique. Therefore, avoiding the AES-GCM accelerator might cause larger overheads in power consumption and resources utilization in the embedded system compared to eight-parallel AES-GCM in Reference [19]. We constructed q=4 parallel structures for GCM, as shown in Figure 7. This combinational architecture is based on four AES subcores and four Ghash subcores. The latter GCTR consists of four KO multipliers. Inside, GCTR (Galois Counter with key K) achieves the confidentiality of data with the block cipher in counter mode, and Ghash realizes high-speed integrity and authentication in the embedded system, which avoids the possibility that the confidentiality approaches cannot be fully protected. In this parallel architecture, the realization of four pipelined AES cores utilize the AES multiplexing structure that was described in Section 4.1, and its input blocks are set with the initial counter block (ICB) and one-increments ($CB_{i}$). Moreover, the plaintext blocks ($P_{i}-P_{i+3}$) are used as the input data blocks XORed with the AES key streams to generate the ciphertext blocks. We assume that the *n* blocks are a multiple of parallelism *q* and that there is no *AAD*; when *n* is not a multiple of *q*, a mask gate is set to append zero blocks to the *q*-mod(n,q) blocks.

In the low-complexity Ghash module using four-parallel KO multipliers, the produced results of $GF(2^{128})$ multiplications are associated with the input parameters of Hash subkey (*H*), which is generated by applying the AES engine to a 128-bit zero block as *H* = Enc(*K*, $0^{128}$). Ghash function addition–multiplication operation is calculated as follows.

(6) $$X_{i}=\left(A_{i}⊕X_{i-1}\right)\cdot H$$

where $A_{i}$ is expressed as the ciphertext block that is input to the Ghash engine and $X_{i}$ is the intermediate variable of hash computation. In addition, the procedures of multiplications and exponentiations over $GF(2^{128})$ are constructed by the irreducible polynomial $g\left(x\right)=x^{128}+x^{7}+x^{2}+x+1$. We note that the related research work indicates that the 10 data blocks are recommended for four-parallel architecture [30]. Then, the $X_{10}$ can be expressed as follows.

(7) $$X_{10}=A_{1}\cdot H^{10}⊕A_{2}\cdot H^{9}⊕\cdots ⊕A_{9}\cdot H^{2}⊕A_{10}\cdot H$$

For improving the pre-calculated term subkey *H* computations, the $X_{10}$ in Equation (7) can be further expressed by using classical squaring method as follows.

(8) $$\begin{array}{c} X_{10}=\left(\left(A_{1}\cdot H^{4}⊕A_{5}\right)\cdot H^{4}⊕A^{9}\right)\cdot H^{2}\\ \;\;⊕\;(\left(A_{2}\cdot H^{4}⊕A_{6}\right)\cdot H^{4}⊕A^{10})\cdot H\\ \;\;⊕\;\left(\left(A_{3}\cdot H^{4}⊕A_{7}\right)\cdot 1\;⊕0\right)\cdot H^{4}\\ \;\;⊕\;(\left(A_{4}\cdot H^{4}⊕A_{8}\right)\cdot H^{2}⊕\;0)\cdot H \end{array}$$

Equation (8) describes that for the four-parallel 128-bit KO multipliers, in the first cycle, four ciphertext blocks $A_{1}$, $A_{2}$, $A_{3}$, $A_{4}$ are multiplied simultaneously by the same subkey $H^{4}$, and the intermediate results are stored in registers, respectively. Similarly in the next cycle, $A_{5}$, $A_{6}$, $A_{7}$, $A_{8}$ data blocks are first XORed with the intermediate results of registers; then, subsequent results are multiplied by the $H^{4}$, $H^{4}$, *1*, $H^{2}$ and overwritten stored in registers, respectively. In the last cycle, $A_{9}$, $A_{10}$, *0*, *0* data blocks are XORed with the register results of the last cycle, then subsequent results are multiplied by the $H^{2}$, *H*, $H^{4}$, *H* and overwritten in registers for generating the integrity Tag.

## 5. SoC Architecture against External Physical Attacks

In this section, we describe the hardware implementation of SoC security architecture with the proposed AES-GCM cryptographic accelerator for preventing the sensitive data leakages and external data tampering attacks. We will also expatiate the dynamic data monitoring mechanism with low performance overhead.

### 5.1. Embedded System Architecture for Security Monitoring

**Figure 8:**
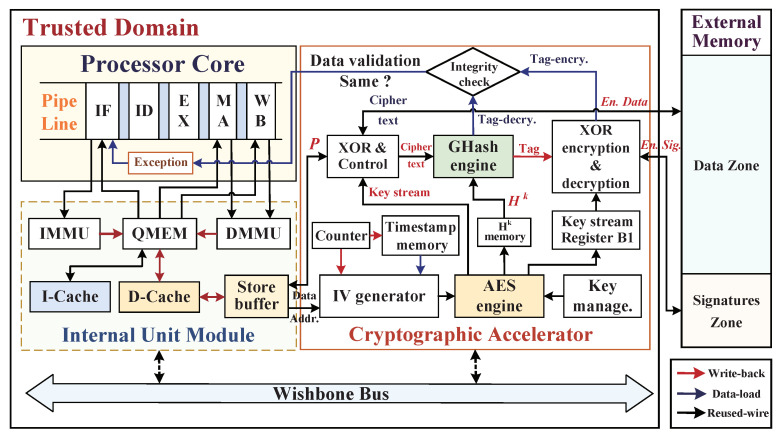
The overall hardware security architecture for dynamic data monitoring with AES-GCM cryptographic accelerator.

Our implementation of target embedded system adopts an open-source reduced instruction set computer (OpenRISC) processor with a Harvard micro architecture, and this softcore processor OR1200 has a five-stage pipeline in the sequential executions, which consists of the instruction-fetching (IF) stage, the instruction decode (ID) stage, the execution (EX) stage, the memory accessing (MA) stage, and the writing back (WB) stage. Moreover, the CPU core can be easily extended with the hardware-assisted cryptographic accelerator through the Wishbone system bus communication protocol. The system bus also connects internal hardware components by using separated data and address bus. Figure 8 shows the overall hardware security architecture that integrates the processor core, cryptographic accelerator, and external main memory, in which hardware accelerator is applied between the store-buffer and external memory to provide data confidentiality and integrity protections during program execution.

In the process of CPU loading/storing the data, first, the CPU sends an effective address for reading/writing data, the data memory management unit (DMMU) translates the effective address into a physical address and sends it to the addressable quick memory (QMEM), and the QMEM judges whether the physical address is within the address range of QMEM—if it is, read and write the specified address directly, and if it is not within the address range of QMEM, send the address to D-Cache. The D-Cache checks whether the target address has been cached, if the D-Cache hit, then it directly sends the corresponding data to QMEM and forwards it to CPU; if the D-Cache missed, it will access the external memory to read and write data through the store-buffer and data WB_BIU modules, where WB_BIU is not shown in Figure 8 for the sake of brevity. Because the external memory is located in the untrusted domain and faces the risk of being attacked maliciously, we set the hardware accelerator to be activated only when D-Cache misses. The Write-Back mechanism of D-Cache plays an important role in achieving low system performance overhead.

The extended hardware-assisted cryptographic accelerator mainly includes the AES engine, Ghash engine, Counter, Key management unit, and Integrity check module. Inside, the AES engine and Ghash engine with four-parallel subcores are utilized to provide confidentiality and integrity protections for data storage off-chip. Moreover, the integrity check module is used to verify the data validation and send normal/abnormal signal to the CPU Exception interrupt module during read-load operation. Timestamps are generated by increasing the counter with one-increment, and the count values are stored in the timestamp memory and associated with the physical addresses of data blocks and as the inputs of the initialization vector (IV) generator. It is noteworthy that the possible situation of the counter overflow will generate a repeated timestamp with previously stored value in the timestamp memory so that the time uniqueness of the key seed cannot be guaranteed and the confidentiality of ciphertext will be reduced; therefore, the sizes of counter and timestamp memory should be configured according to the application requirements for avoiding the counter flow. In order to ensure the security of timestamps, the timestamp memory is located in the on-chip trusted domain on which stored timestamps are considered as immune to the above-mentioned external attacks. We distinguish the write-back and the data-load procedures with red arrows and blue arrows, respectively, while the reused signal wires with black arrows are utilized in both encryption and decryption processes. In order to improve the confidentiality of dynamic data, we adopt the data blocks’ (plaintext) corresponding physical address as part of IV seed for ensuring the spatial uniqueness of key stream, and the timestamp is utilized to ensure the time uniqueness of IV seeds. Hence, this encrypted method has the superior capabilities in resisting spoofing attack, relocation attack, and replay attack.

### 5.2. Data Blocks Write-Back Procedure of Memory Access

**Figure 9:**
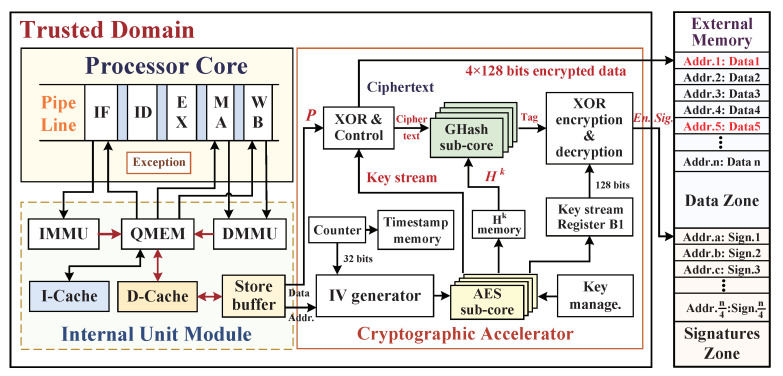
The embedded system details of D-Cache data blocks write-back procedure.

The innovation of our hardware security mechanism is integrating a high throughput four-parallel AES-GCM cryptographic accelerator, which can effectively reduce the encryption- and decryption-required clock cycles while processing large numbers of data. In normal operations, the working process in the embedded system in protecting the dynamic data is divided into two stages: the calculated data from D-Cache write back to the external memory, and the data from external memory are loaded into the on-chip D-Cache for awaiting the processor’s next computing. Moreover, the locality principle of D-Cache mapping main memory plays an important role in decreasing external memory accessing times and performance overhead caused by data integrity checking. Figure 9 shows the operation details of dynamic data writing back to the external memory; we consider the size of data-protecting granularity is compatible with AES-GCM accelerator that is an integer multiple of 128 bits, so we configure the lengths of plaintext and ciphertext blocks as 128 bits (four 32-bit data sub-blocks), and D-Cache line size is also set as 128 bits (16 types), so that the data blocks can be expressed as *n*×128 bits, where *n* denotes the number of 128 bits data blocks from D-Cache. The physical addresses of external memory are aligned to four bytes, and the lower two bits are fixed to 2’b00 for 4 sub-blocks offsetting in each 128-bit storage block.

#### 5.2.1. Hash Subkey $H^{k}$ Pre-Calculation

We have allocated the four-parallel hardware architecture with the Hash subkeys $H^{k}$ (*k* = 0, 1, 2, 4) for implementing high-efficiency multiplications in the Ghash engine, in which the hash subkey exponentiations k=2 and k=4 require squaring operations, and it is known that the squaring operation in binary extension fields leads to a linear structure. In the hardware realization of hash subkey $H^{k}$ pre-calculation process, three extra multipliers are divided into two levels that are configured between the AES engine and the $H^{k}$ memory; among it, two parallel multipliers at the first level connect the outputs of four-parallel AES sub-cores, and the other one multiplier at the second connects the two multiplier outputs of the upper level. Then, the subkey *H* can be derived directly by applying an AES sub-core to a 128-bit zero block, and the hash subkey exponentiations $H^{2}$ and $H^{4}$ are obtained from the outputs of the multiplier at the first and second levels, respectively. Finally, these pre-calculated hash subkeys of *1*, *H*, $H^{2}$, $H^{4}$ are stored in the $H^{k}$ memory for the Ghash engine subsequent computations. Compared with many cascaded squaring operations, this complexity-reduction technique uses four-parallel multiplexing method decreasing the number of gates and has the lowest critical path delay while its hardware complexity is reasonable.

#### 5.2.2. Dynamic Monitoring Mechanism with D-Cache

**Figure 10:**
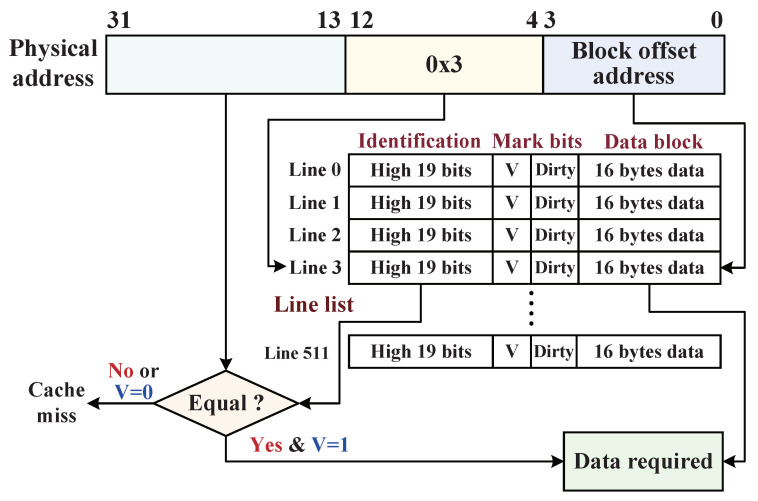
The internal implementation structure of D-Cache with the size of 8-KB.

As mentioned above, D-Cache plays an important role in dynamically activating the data monitor for data encryption and decryption. The implementation structure of D-Cache is shown in Figure 10. When the CPU requests to write back a 32 bits data sub-block to external main memory, the 32-bit physical address ($ADD_{phy}$) of data sub-block in the address bus consists of three parts, its high 19-bit $ADD_{phy}$[31:13] is compared to the high 19-bit identification tag in the indexed cache line appointed by the $ADD_{phy}$[12:4] if their comparative result is equal; meanwhile, the mark bit of *Validity* (V) in cache line is “1”, which indicates a D-Cache hit, the physical address can exactly access its target address according to $ADD_{phy}$[3:0] (block offset address), and the data sub-block of target address is overwritten by the write-back sub-block. Once the cache line completes an overwriting and has changed (where main memory not updated), its *Dirty* bit turns “0” to “1”. Otherwise, D-Cache misses when their comparative result is unequal, which indicates the write-back target address was not cached in D-Cache, or the physical address is allocated to the invalid cache line (V = “0”), in which the original data block is invalid. Then, D-Cache directly flushes the free cache line with four data sub-blocks from the target addresses on the main memory with the direct mapping method, and CPU completes the overwriting operation, line *Dirty* bit marked with “1”. Finally, D-Cache synchronizes the data blocks (*Dirty* marked with “1”) to the main memory with encryption protections at store status. It is noteworthy that the encrypted data blocks are the D-Cache 128 bits line blocks and their Dirty is marked with “1”, to stay consistent with the main memory.

#### 5.2.3. Write-Back Procedure of D-Cache Data Blocks

When D-Cache addressing misses, the D-Cache prepares to write back the data blocks to the memory addresses, while the cryptographic accelerator is activated immediately. Algorithm 1 describes the encryption procedure of writing back the data blocks into the external memory, and the corresponding hardware details of this procedure are shown in Figure 9. Afterward, the appended cryptographic accelerator starts obtaining the plaintext data blocks and their corresponding storage addresses from the D-Cache module. The counter is configured to generate the count values with one increment, and as the timestamps are one-to-one associated with the data blocks physical addresses, the timestamps are stored in on-chip timestamp memory in the trusted domain. Then, the IV seeds are composed with input—both the first 32-bit physical address and the 32-bit timestamps through the IV generator—and one 128-bit IV seed is appended with 64 bits zero block (IV_seed[95:32]) between the high 32-bit physical address (IV_seed[127:96]) and the low 32-bit timestamp (IV_seed[31:0]). To better match the four-parallel AES-GCM sub-cores hardware architecture and improve its processing efficiency, *n* data blocks are assigned into $\frac{n}{4}$ rounds multiplied by four data blocks, and 4×128 bits data plaintext blocks with the 16 related physical addresses are participated in every cryptographic computing operation. In the case that *n* is not a multiple of four or 1≤n≤3, zero blocks are appended to the remainder computing sub-cores.
**Algorithm 1** Write-back operation of data blocks being stored into external main memory**Inputs:***Data*, *Address***Outputs:***Timestamps*, *Ciphertext*, *Signature*  1: *Data* ← set of data blocks to write back ${data}_{i}$, 1≤i≤n.  2: *address* ← set of memory physical ${address}_{d}$, 1≤d≤n.  3: **pre-calculation**
*H* = $AES_{k}\left(0^{128}\right)$, squaring operations, hash subkeys *1*, *H*, $H^{2}$, $H^{4}$ are pre-stored in $H^{k}$ memory;  4: D-Cache miss, mapping address to physical address;  5: **begin** inputting four physical address blocks, counter generates timestamps (Ts), Ts++, are stored in Ts memory;  6: IV_seed = {*address* [127:96], $0^{64}$, *timestamp* [31:0]};  7: **repeat**: the four IV seed blocks are generated;  8: Using AES engine to generate *key_stream*, and storing the first block *key_stream_B1* in register B1;  9: *Ciphertext* = *Data* XOR *key_stream*, **then** input ciphertext blocks to the GHash engine;10: **output**: ciphertext blocks at another branch path are stored into the data zone of external memory;11: **until** ciphertext blocks are computed in the GHash engine, *signature* = *Tag* XOR *key_stream_B1*;12: **output**: integrity digital signature is stored into the signatures zone of external memory;

The key management unit is used to provide a 128-bit initial key for AES engine key expansion process and rolled-pipelined 10-round operations. While the four-parallel AES engine sub-cores outputting 4×128 bits encrypted key stream to the XOR and Control module, we select the 128-bit first block key stream (B1) of AES engine sub-cores as the subsequent XOR cryptographic operand, which is temporarily stored in the key stream register B1 to await the accomplishment of Ghash engine computations. This method not only solves the special 1≤n≤3 situations well, but also does not cause extra hardware consumption in the implementation of four data blocks mapping to the same encrypted digital signature. Then, the four ciphertext blocks are generated through the four input data blocks XORed with the key stream. In a branch path, the ciphertext in the format of 4×128 bits encrypted data blocks are stored in the data zone of target external memory, which satisfies the confidentiality requirement against sensitive data leakages. For the external memory data zone and signatures-zone-related configuration, we set the bit widths of the storage units as 16 bytes, and a storage unit corresponds to a physical address with step 4 (lower 2 bits are fixed to 2’00); therefore, the four encrypted data blocks occupy *Addr.1* to *Addr.4* addresses in the data zone. In another branch path, these ciphertext blocks are also input into the GHash engine that contains four-parallel KO multipliers, and a 128-bit authentication tag is computed for ensuring that the integrity of ciphertext data are not maliciously tampered with. Ultimately, the authenticated Tag is XORed with the previously stored key stream B1 to generate a encrypted digital signature, which is stored into the *Addr.a* of signatures zone; this final XOR operation can establish the correspondence between one digital signature and four ciphertext blocks.

### 5.3. Data Blocks Read-Load Procedure of Memory Access

**Figure 11:**
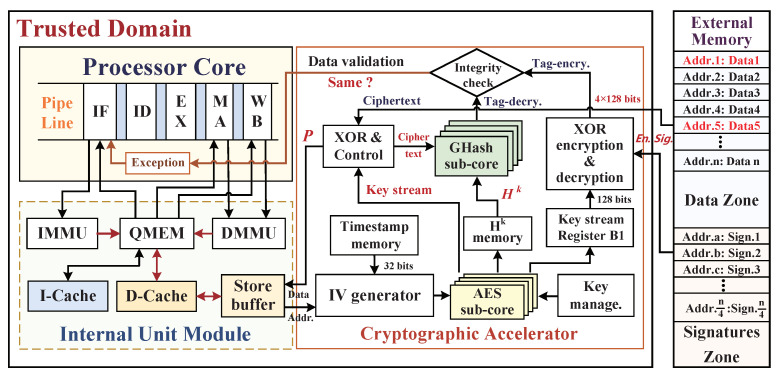
The hardware monitoring architecture details of loading the ciphertext data blocks.

In the procedure of read-load operation from the external main memory, the request physical address of CPU is first asserted and sent to the D-Cache. If the $ADD_{phy}$[31:13] is equal to the high 19-bit identification tag of the appointed cache line, while the line mark bit of V is “1”, then D-Cache hits, the cached data sub-block corresponding to the physical address, is sent to the processor directly. Otherwise, if D-Cache misses, the D-Cache will read-load the data sub-blocks from external main memory, and their physical addresses will be first asserted and sent to cryptographic accelerator and external main memory. It is noteworthy that the hardware monitor architecture is designed for four-parallel blocks synchronously processing, and four ciphertext blocks and a corresponding digital signature are read-loaded to the hardware monitor even if CPU sends a read request $address_{d}$ of one ciphertext block; meanwhile, the physical addresses corresponding to four ciphertext blocks are buffered immediately in D-Cache through the Wishbone address bus. This operation method has adapted the locality principle of D-Cache mapping memory, which can increase the locality intensity on the D-Cache hit ratio and its access effectiveness for improving the system performance. According to the input four physical address blocks, timestamp memory pops out the buffered timestamps, which correspond to the physical address blocks as described in the above write-back operation. Algorithm 2 describes the read-load operation of data blocks from the external main memory, and the corresponding hardware architecture details of the read-load procedure are shown in Figure 11.
**Algorithm 2** Data-load operation with hardware security checking from external memory**Inputs:***Address*, *Ciphertext*, *Signature***Outputs:***Data*, *Exception*  1: *Data* ← set of data blocks to write back ${data}_{i}$, 1≤i≤n.  2: *signature* ← Integrity ${signature}_{s}$ set of data blocks and corresponding addresses, $1\leq s\leq \frac{n}{4}$.  3: assert the target address, map address to physical address;  4: **assign**
${address}_{i}$ ← q×mod(d,q)+1, i = 1, i++, 1≤i≤4,       *signature* ← mod(d−1,q)+1, where q=4;  5: **Input** four physical address blocks and a corresponding signature to cryptographic accelerator;  6: **begin** timestamps memory pops four timestamps, Ts++;  7: IV_seed = {*address* [127:96], $0^{64}$, *timestamp* [31:0]};  8: **repeat**: the four IV seed blocks are generated;  9: Using AES engine to generate *key_stream*, and storing the first block *key_stream_B1* in register B1;10: *Data* = *Ciphertext* XOR *key_stream*, **then** *Ciphertext* = *Data* XOR *key_stream* input to the GHash engine;11: **until** ciphertext blocks are computed in the GHash engine, the authenticated Tag of *Tag-decry.* is output;12: *Tag-encry.* = *Signature* XOR *key_stream_B1*13: **if**
*Tag-decry.* = *Tag-encry.* **then**     *Exception* = NULL    /* integrity valid */    **else** *Exception* = assertion;    /* integrity invalid */

In the cryptographic accelerator, the IV generator module outputs four IV seed blocks into the AES engine, and the four-parallel sub-cores in AES engine are utilized again to generate the key stream. The first block 128-bit key stream (B1) is stored in a register. In the meantime, the read-load operation feeds back the encrypted data blocks and the corresponding encrypted signature from the external memory. The plaintext (*P*) blocks are obtained by employing ciphertext blocks XORed with the new key stream and next input to the store buffer. Furthermore, the created plaintext blocks continue to XOR with the new input 4×128 bits key stream under inside control logics, and then the GHash engine computes the authenticated Tag in the decryption period (Tag-decry). In addition, the authentication Tag in encryption period (Tag-encry) is calculated by utilizing the input digital signature XORed with the key stream (B1) in configured XOR encryption and decryption module. Finally, the decrypted Tag-decry will be compared with the previous encrypted Tag-encry in the integrity checking module, and the exception signal of data invalidation will be sent to the processor exception unit if any violation is detected.

## 6. Experiments and Results

Experiments and results are presented in this section to expatiate the effectiveness and performance characteristic of the proposed cryptographic accelerator. We first describe the setup of experimental setup and verification platform. Then, the system performance overhead, security capability, and processing efficiency are evaluated. Finally, the resource consumption of SoC is presented in detail.

### 6.1. Experimental Setup

We implement the hardware-assisted AES-GCM cryptographic accelerator into open-source processor OR1200 embedded system for guaranteeing intensive data processing security, in which the basic frequency of scalar Reduced Instruction Set Computer (RISC) processor is set @100 MHz, and clock cycles satisfy the synchronization with the cryptographic accelerator. In the processor component configurations, the independent I-Cache and D-Cache modules both support the different size configurations of 2-KB, 4-KB, 8-KB, and 16-KB, and we first configure the softcore processor with a typical configuration of 8-KB I-Cache and 8-KB D-Cache, whose internal structures consist of 512 cache line blocks. We designed the whole embedded system with the cryptographic accelerator in Verilog hardware description language (HDL) and performed the logic synthesis and implementation with Xilinx ISE Design Suite 14.7. This SoC hardware architecture integrating cryptographic accelerator is finally evaluated on a Xilinx Virtex-5 FPGA platform, and the GNU Cross Compilation Toolchain or32-elf-gcc matching with OR1200 core matched is utilized to generate CPU execution codes. We employed both 18-MB Synchronous Static Random-Access Memory (SSRAM: IS61LPS51236A) and 32-MB Synchronous Dynamic Random-Access Memory (SDRAM) as the external memory of the FPGA evaluation platform. The external memory should be initialized during the system initialization stage (Boot Process): first, the bitstream is duplicated from the external flash memory to FPGA at power-up; meanwhile, the RAM blocks of inner FPGA are instated with the bootloader (U-Boot), which duplicates the kernel from flash memory to the external RAM Memory and establishes the mapping of memory space, and then loader branches to the proper RAM area of kernel and system boots to execute programs.

### 6.2. Performance Overhead Evaluation

While the embedded system executing program operational codes and activating the hardware security monitor, the proposed cryptographic accelerator will inevitably result in a performance overhead to processor. In the system architecture design, we have made some efforts to reduce system performance overhead during the authenticated encryption procedures, for example, the design optimizations of the D-Cache configuration and four-parallel structure to reduce the performance overhead. In the experiments of SoC performance evaluations, ten various scales of the embedded benchmarks from Mibench suite [31] are applied to perform the realistic application workloads. These selected benchmarks are first compiled by using the GNU Cross Toolchain or32-elf-gcc and downloaded onto the FPGA platform for processor executing. Moreover, the number of total instructions of each benchmark are counted. Considering that the hit rate of I-Cache and D-Cache may influence the performance overhead of SoC, we first configured the I-Cache and D-Cache both with 8 KB, and utilized the or1ksim [32] tool as system simulator to record the I-Cache and D-Cache hit rates. Therefore, we can calculate the average cycles per instruction (CPI) of processor with and without integrating AES-GCM cryptographic accelerator to evaluate the performance overheads under the different benchmarks.

These SoC performance evaluation results are shown in Table 1; these experimental data indicate that the SoC average performance overhead is 2.65%, ranging from 1.08% (quicksort) to 5.03% (OpenECC). Benchmark of AES has the highest D-Cache write-hit and read-hit rates and beyond 99.5%. The average hit rates of 8-KB I-Cache and 8-KB D-Cache have both exceeded 98%, which are plays an important role in reducing the performance overhead caused by cryptographic accelerator. By carefully analyzing the results, the indicator CPI trends vary with the number of benchmark instructions, which reflects a larger proportion of the external memory access instructions in the binary operational code, when more memory access instructions appearing in a benchmark CPI will be higher, such as the benchmarks of the OpenECC, basicmath, and patricia, which executing a large number of external memory access operations to store the temporary data, thus having the higher CPI data than other benchmarks. We can also learn that the processor performance overhead depends on the responding speed of the external memory; therefore, we employed the SSRAM as the external memory, which provides a faster memory access speed than other types of memory to reduce the performance overhead.

**Figure 12:**
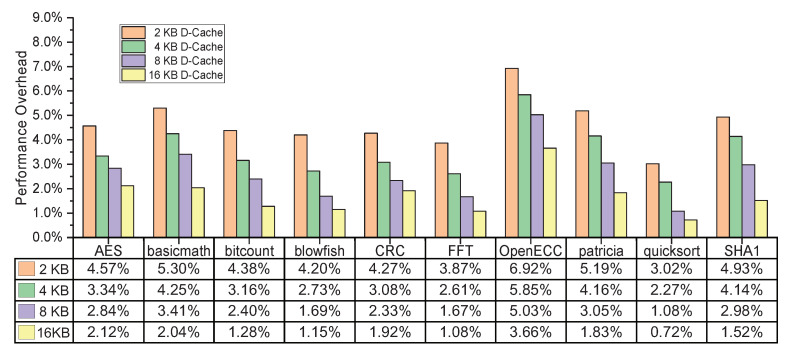
The performance overheads of the selected benchmarks under different sizes of D-Cache.

To further explore the influences of D-Cache hit rates on performance overhead, we continue the evaluation experiments of performance overhead with the remaining 8-KB I-Cache unchanged; the size of the D-Cache is re-configured as 2-KB, 4-KB, and 16-KB. Figure 12 shows the performance overheads of the selected benchmarks based on the different sizes of D-Cache. Due to the enlargements of D-Cache addressing spaces, the performance overhead decreases with the raise of the D-Cache hit rates. After we configured the D-Cache with a size of 16-KB, the performance overhead of embedded system had a significant reduction and ranges from 0.72% to 3.66%. The mechanism for this effects is that when the hit rates of D-Cache increase, the number of times of the cryptographic accelerator being invoked will decrease, such that the encryption, decryption, and authentication operations will incur additional clock cycles. In summary, for the implementation of applied embedded system, we can configure an appropriate size of D-Cache to reduce the miss rate, thereby helping the target SoC to decrease performance overhead.

### 6.3. Security Capability Evaluation

We implemented the monitoring mechanism of cryptographic accelerator by considering two threats: information leakages and tampering attacks. While activating the hardware security monitor, AES engine generates the key stream according to physical addresses and timestamps, the ciphertext blocks are easily obtained through the simple XOR operations between plaintext blocks and key stream at run-time. Even though physical addresses and ciphertext are intercepted by adversaries during ciphertext blocks being stored in the memory, the four unknown corresponding timestamps, 128 bits AES cipher key, and 128×4 bits key stream make it impossible for attackers to reversely derive the desired plaintext blocks in limited time. This confidentiality protection method has a good robustness by resisting bus monitoring and offline analysis attacks. Moreover, the plaintext blocks are also input to the GHash engine and generate an integrity Tag; then the Tag XORed with a block of key stream creates an associated digital signature, which is stored in memory signature zone for reducing on-chip storage overhead. Whenever the ciphertext blocks and the signature blocks are maliciously tampered with, this will be detected rapidly in Tags integrity checking operations. Therefore, the combination of confidentiality and integrity protections can provide a high-level security for dynamic data processing in SoC against information leakages and external tampering attacks.

To confirm the effectiveness of hardware security monitoring, we configured the OR1K debugging system that is combined with the Joint Test Action Group (JTAG)-TAP module and Advanced Debug Interface (ADI). This debugging system acts as the interface to communicate directly with the CPU and Wishbone system bus, so that we could start and break the executions of program and read and write CPU internal registers by accessing the CPU. We performed the data tampering experiments at run-time based on the debugging system. During the program execution, the specific situations of D-Cache misses are created artificially, and three types of data tampering attacks are introduced into the data path bus to modify the ciphertext sub-blocks in external memory, respectively. Table 2 depicts the security capability tests of SoC on integrating cryptographic accelerator under the different data-tampering attacks. The exception results of integrity error can be printed and displayed through error_log files in the upper machine. We can find that the hardware monitor integrity verification can detect any malicious tampering behaviors of data blocks. The CPU stall signal can suspend the pipeline stages and pause the program execution and data processing and transfer the processor core to a secure mode. All the pipeline stages are “frozen” until the integrity checking passes and the CPU stall is de-asserted.

### 6.4. Efficiency Evaluation of Encryption and Decryption

For the performance overhead evaluations of the embedded system, we have presented the experimental results of benchmarking the processor performance with different CPIs. However, the evaluation of computing the efficiency of the cryptographic accelerator is not presented with the available reference data. Due to these uncertain activating operations, clock cycles are considered as the measurement indicator of processing efficiency, and we first make sure that start measuring the performance penalty when D-Cache misses. Specifically, we implemented 4×128 bits plaintext data blocks to measure the clock cycles of encryption and decryption operations. The timestamp counts and storage need four clock cycles, and AES engine takes 12 clock cycles to complete the substitution and permutation operations. The clock cycles for GHash engine with four-parallel KO multipliers is represented as $\frac{n}{q}+\log_{2}\left(q\right)$, where *q* is a parallelism constant of four, and *n* is the number of 128-bit data blocks, then three clock cycles are needed to complete multiplication calculations. It is noteworthy that the clock cycles of encryption and decryption do not include the Hash subkey $H^{k}$ pre-calculations, logic XORs, etc., and input and output data clocks, in which Hash subkeys are pre-calculated and stored in the $H^{k}$ memory before encryption operation.

**Figure 13:**
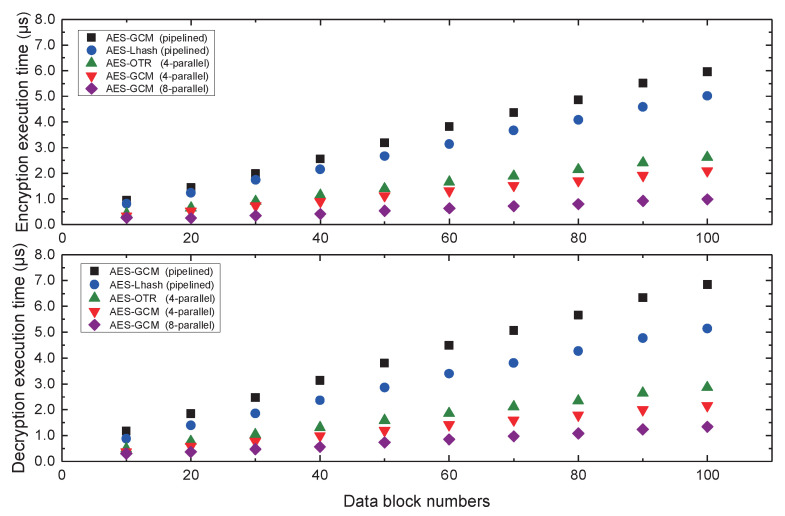
The processing performance of the four-parallel cryptographic accelerator compared to the other security architectures.

Computing one round of encryption and decryption in embedded system may not comprehensively measure the processing efficiency of the four-parallel AES-GCM hardware accelerator. We implemented the multiple data blocks encryption and decryption experiments, and provided 10–$10^{2}$ plaintext data blocks to verify accelerator high-throughput performance. Figure 13 has illustrated the processing performance of 4-parallel AES-GCM hardware accelerator, which is compared to the other similar security mechanisms as @100 MHz frequency. We note that the eight-parallel high-performance AES-GCM architecture proposed in Reference [33] has the highest efficiency in both encryption and decryption computations with the highest hardware complexity. Conversely, the single pipelined AES-GCM architecture takes the most time cycles to complete the computation on the authenticated encryption [34], even more than the competitive AES combined with the light-weight Hash (LHash) algorithm proposed in the Reference [35]. The SoC performance evaluations indicate that the data-processing efficiency of the four-parallel cryptographic accelerator is around 3 times that of the pipelined AES-GCM construction, which achieves a trade-off between high-throughout and hardware complexity. According to the equation of throughput calculation, $Throughput=Frequency\times 128\times \frac{\mathrm{frames}}{cycles}$, its data processing throughput speed can reach 14.93 Gbps @100 MHz, whose authenticated efficiency is better than the other efficient four-parallel AES-OTR hardware architecture presented in Reference [36].

### 6.5. Hardware Implementation Overhead

The advantages of hardware cryptographic accelerator are its high efficiency in authenticated encryption with minimal requirement of clock cycles. This security monitor inevitably increases the hardware resource consumption and chip area. The register-transfer level (RTL) SoC architecture is synthesized, implemented, and verified on a Xilinx Virtex-5 (XC5VLX220T) FPGA development platform, and its hardware resource consumption is shown in Table 3. The occupied slices for the proposed cryptographic accelerator is around 69.2% of total SoC because of its four-parallel high-efficiency hardware structure. In the experimental platform, an 8-bit counter is utilized to generate the 32-bit timestamps for the purpose of decreasing the requirement of on-chip memory, which leads to it occupying about 19.8% of memory resources. The optimal hardware realizations of AES engine, S-Box complexity with composite field arithmetic, have saved a large of resource consumption. By comparing our results of four-parallel AES-GCM to the previous works, we show that our hardware overhead is lower than that of Reference [37] with better security capability. According to the hardware resource consumption on the FPGA platform, we can conclude the chip size of our SoC after tape-out is smaller than integrating the other four-parallel architecture [37], the eight-parallel architecture [19], and the four-parallel AES-OTR architecture [36], but it is accompanied inevitably by a larger resource consumption than integrating the pipelined AES-GCM architecture [34] and the pipelined AES-LHash architecture [35]. Our OR1200 core is a five-stage pipeline softcore processor; for the other embedded processors, like the RISC-V softcore processor, this cryptographic accelerator can be easily transplanted and may have less of an impact on performance overhead.

## 7. Conclusions

This paper presents an integrated high-performance cryptographic accelerator to protect dynamic data security in an embedded system. The accelerator architecture employs a four-parallel AES-GCM hardware structure to provide authenticated encryption for preventing sensitive information leakages and data tampering attacks caused by the off-chip physical attacks. It helps the embedded system to build trustworthiness to guarantee the characteristics of data confidentiality and integrity, which is an effective method to generate integrity digital signature for security verification. We performed performance overhead and processing efficiency evaluations; the results showed that the cryptographic accelerator achieves a high-efficiency encryption processing while maintaining a low performance overhead. Its average performance overhead reduces to as low as 2.65% on the typical 8-KB I/D-Caches. The security capability evaluations confirm the monitoring effectiveness of cryptographic accelerator against both three types data tampering attacks. Finally, the SoC security architecture satisfies a good tradeoff between performance overhead, processing efficiency, and hardware overhead.

## Figures and Tables

**Table 1:** Performance overhead of processor configured with cryptographic accelerator (8 KB I-Cache and 8 KB D-Cache).

Benchmark	Total Instructions	I-Cache Hit	D-Cache Read Hit	D-Cache Write Hit	CPI without AES-GCM	CPI with AES-GCM	Performance Overhead
AES	22,170	98.97%	99.84%	99.65%	3.52	3.62	2.84%
basicmath	26,515	98.08%	98.63%	98.57%	2.64	2.73	3.41%
bitcount	19,684	97.95%	96.47%	95.92%	1.67	1.71	2.40%
blowfish	19,128	97.67%	97.70%	97.44%	3.54	3.60	1.69%
CRC	18,941	99.49%	98.38%	97.65%	1.72	1.76	2.33%
FFT	13,506	95.62%	98.45%	98.16%	2.39	2.43	1.67%
OpenECC	56,313	99.14%	99.12%	98.58%	3.18	3.34	5.03%
patricia	23,130	97.68%	97.06%	96.39%	1.64	1.69	3.05%
quicksort	6707	99.12%	98.89%	98.67%	1.86	1.88	1.08%
SHA1	20,455	98.65%	99.32%	99.21%	2.35	2.42	2.98%
Average	-	98.24%	98.39%	98.02%	2.45	2.52	2.65%

**Table 2:** Security capability tests of SoC on integrating cryptographic accelerator under different data-tampering attacks.

Tampering Attacks	Spoofing Attack	Relocation Attack	Replay Attack
Approaches	Write addr.: 0x00002016 Read addr.: 0x00002016	Write addr.: 0x00002016 Read addr.: 0x00002012	T8: 32’h2c9340b4 T4: 32’h4f5c1a08
Data Tampering	32’h68e5a4c5 32’h68e50000	32’h364c91f8 32’hf10d4a12	32’h2c9340b4 32’h4f5c1a08
Exception	Integrity Error	Integrity Error	Integrity Error

**Table 3:** The SoC hardware resource consumptions on Virtex-5 FPGA chip.

Slice Logic Utilization	SoC	Processor Core	AES-GCM
Slice registers	6518	1864	4923
Slice LUTs	16,610	5547	11,956
Occupied slices	10,637	3262	7512
BlockRAM/FIFO	58	13	42
BUFG/BUFGCTRLs	7	1	3
